# Bioactive Compounds Isolated from Marine-Derived Microbes in China: 2009–2018

**DOI:** 10.3390/md17060339

**Published:** 2019-06-06

**Authors:** Weiwei Sun, Wenhui Wu, Xueling Liu, Diana A. Zaleta-Pinet, Benjamin R. Clark

**Affiliations:** School of Pharmaceutical Science and Technology, Health Science Platform, Tianjin University, 92 Weijin Road, Tianjin 300072, China; sww123hi@163.com (W.S.); wuwh7382@163.com (W.W.); 13207507582@163.com (X.L.); diana.zaleta-pinet@outlook.com (D.A.Z.-P.)

**Keywords:** marine-derived microbes, endophytic fungi, drug discovery

## Abstract

This review outlines the research that was carried out regarding the isolation of bioactive compounds from marine-derived bacteria and fungi by China-based research groups from 2009–2018, with 897 publications being surveyed. Endophytic organisms featured heavily, with endophytes from mangroves, marine invertebrates, and marine algae making up more than 60% of the microbial strains investigated. There was also a strong focus on fungi as a source of active compounds, with 80% of publications focusing on this area. The rapid increase in the number of publications in the field is perhaps most notable, which have increased more than sevenfold over the past decade, and suggests that China-based researchers will play a major role in marine microbial natural products drug discovery in years to come.

## 1. Introduction

Natural products continue to be a major source of new compounds in the development of small-molecule drugs, whether that is directly, semi-synthetically, or as a source of inspiration or chemical scaffolds [1,2,3,4,5]. Recent reports state that drugs derived from natural products account for more than 40 billion USD in the market, not including the non-pharmaceutical uses of biologically active polyketides and terpenoids used in agriculture or aquaculture: where the markets are estimated to be worth 17 billion USD and 12 billion USD, respectively [6].

Microbial natural products are not only the most reported for their structural novelty, but they also have a wide array of biological properties with targets that are involved in numerous diseases, such as antibiotics, immunosuppressants, anti-diabetics, hormone (ion-channel or receptor) antagonists, anti-cancer drugs, and even as agricultural agents [7]. In spite of yielding great results, there are several challenges still remaining natural products research. One of the main challenges is the low percentage of microbes that can be readily cultured under laboratory conditions. It is estimated that one gram of fertile soil contains around 107 bacterial types, 105 actinomycetes spores, and 102 fungal spores [8], and we are only able to grow 5% of those microorganisms. Another challenge is the concentration of the desired metabolites in the laboratory cultures: microbial extracts are complex mixtures that can make the isolation and elucidation of microbial natural products a slow and tedious process. New techniques are being developed to address these issues, such as new methods for the isolation and culturing of bacteria, or the extraction and sequencing of metagenomics samples from soil or water samples [9], OSMAC strategies [10], genome mining [11], and metabolomic methods [12,13].

The help of these new technologies has granted us access to new microbiota, and it estimates that we are now able to analyze the genomes of at least 75% of the existing microbiota within a soil sample [8]. It is reported that, over the past three decades, 60% of the new anticancer agents and 75% of new antibiotics can be linked to NPs or their derivatives [8], and it seems clear that, despite the long history of research in the field, both microbes and the marine environment are still capable of yielding structurally novel compounds for the drug discovery process [14].

China offers an enormous potential resource of new microbial strains for chemical investigation. Chinese natural products have a long history, with medicinal traditions dating back thousands of years. Modern studies by Chinese researchers began in the 1930s, which focused on the chemistry of plants that are used in traditional medicinal preparations, and continue to be dominated by research into the natural products of plants [15]. The award of the 2015 Nobel Prize in Medicine to Tu Youyou for her discovery of the antimalarial agent artemisinin [16] illustrates the potential of Chinese natural products research to produce truly significant biologically active compounds.

However, it is only in the past few decades that China-based natural products researchers have turned from traditional sources of natural products discovery, such as plants, towards microbial and marine drug discovery research. In China, several groups are in the process of evaluating the potential of microbial natural products, for with its great land-mass, wide array of micro climates, and vast oceans, China may possess a unique microbial diversity and corresponding potential for novel chemistry [17,18,19]. High-profile research is being conducted into the pursuit of natural products that are isolated from marine bacterial sources [20,21,22], fungal endophytes from unusual sources, such as mangroves [23], in addition to a burgeoning crop of researchers in the field of natural products biosynthesis [24]. The oceans are one environment that has yielded particularly promising results: marine natural products have yielded diverse compounds over the past 20 years [25,26], many of which have shown potential as leads for drug development [27]. In the current review, we focus on the research of China-based research groups on the isolation of biologically active metabolites from marine-derived fungi and bacteria. Numerous examples of the bioactive molecules that were isolated from these sources over the past ten years are given. The origin of the strains, the diversity and biological properties of the compounds produced, and the numbers and venues for publication of this research are also summarized (Supplementary Table S1).

## 2. Polyketides

### 2.1. Bacterial Polyketides

**Figure 1:**
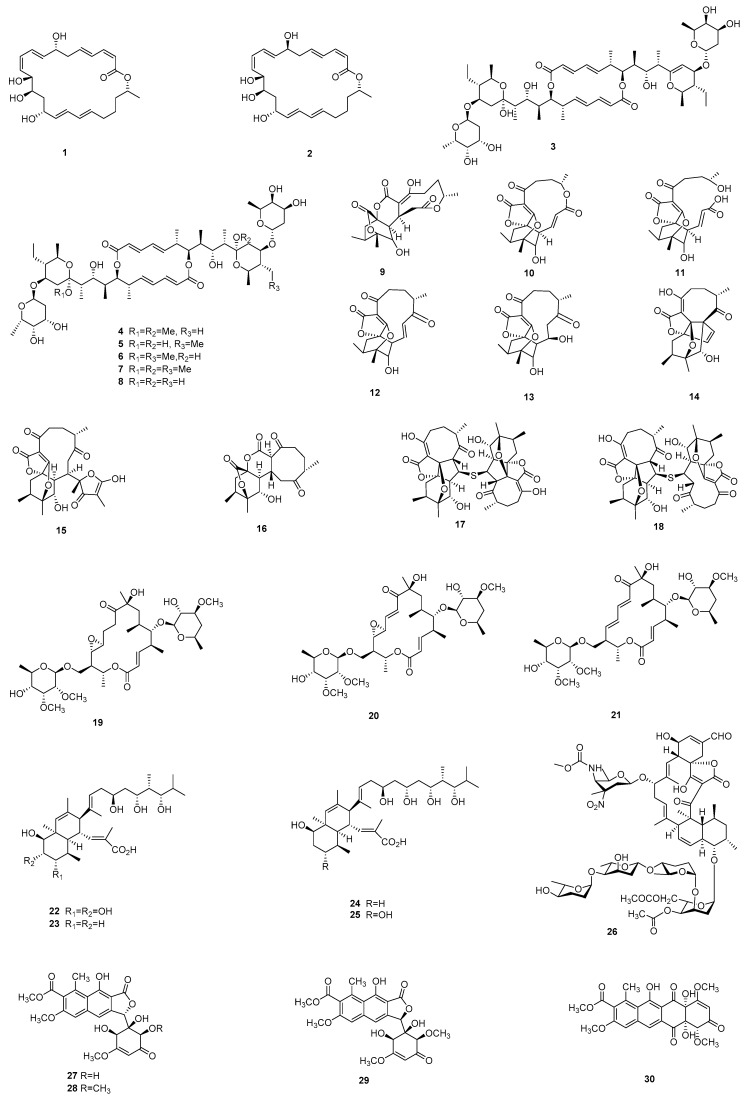
Selected polyketides **1**–**30** isolated from marine-derived microbes.

**Figure 2:**
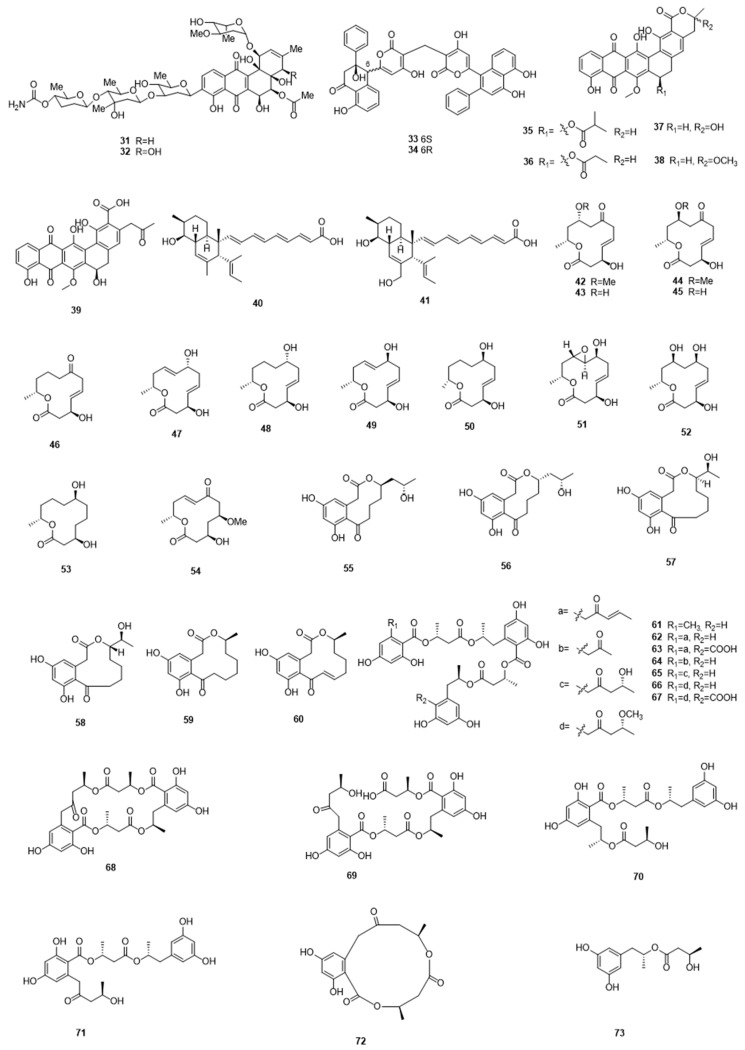
Polyketides **31**–**73**, isolated from marine microbes.

**Figure 3:**
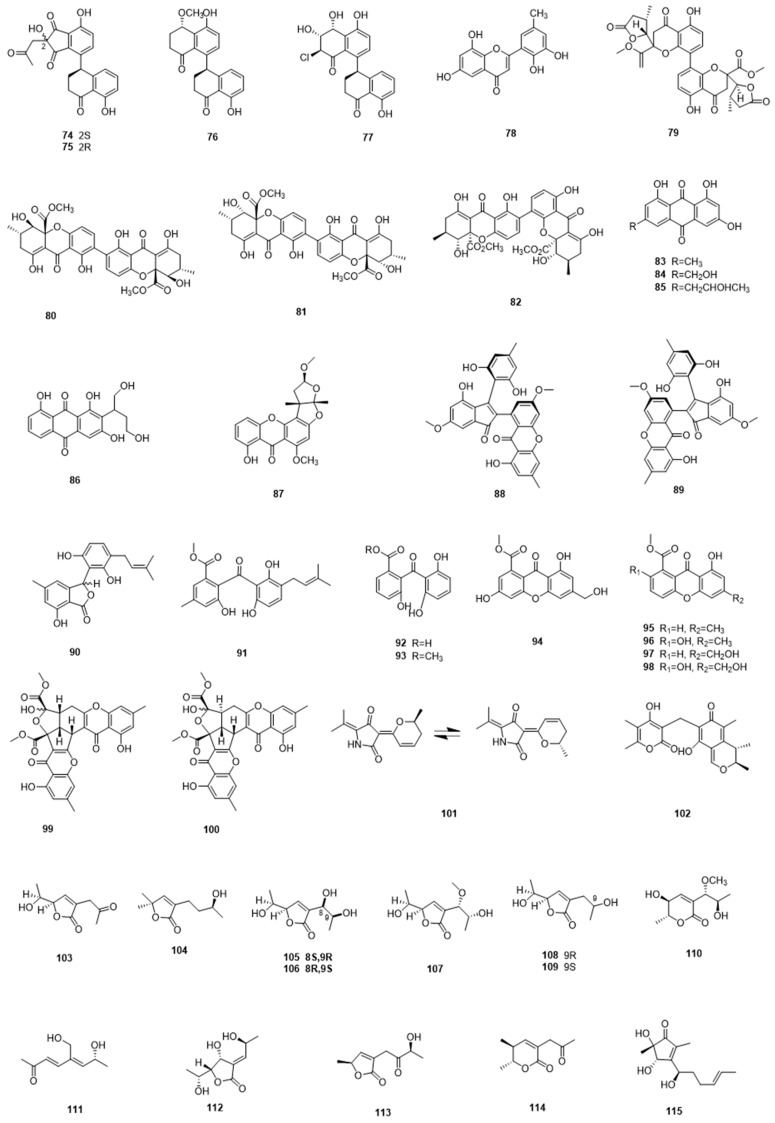
Polyketides **74**–**115** isolated from marine microbes.

Bacterial polyketides mainly consist of two major structural classes, according to their mode of biosynthesis: one group of polyketides is formed by modular polyketide synthases that produce polyketides tending to be linear structures or large macrolactone rings, with frequent alcohols, methyl branches, etc., while another group of polyketides, which are produced by iterative polyketide synthases, tend to have condensed aromatic structures [28]. Both classes are well represented in the literature from China (Figure 1 Figure 2 Figure 3). Most of the polyketides from the first structural class exerted significant antimicrobial activities, and they can be exemplified by the macrolactins isolated by Gao *et al.*, including macrolactin V (**1**), a new 24-membered macrolide, along with macrolactin S (**2**), a known macrolide, from the marine bacterium *Bacillus amyloliquefaciens* SCSIO 00856. Antimicrobial testing showed that macrolactin V possessed potent antibacterial activity against *E. coli*, *Bacillus subtilis*, and *Staphyloccous aureus*, with an MIC value of 0.1 mg/ml [29].

As with terrestrial microbes, *Streptomyces* species isolated from marine microbes continue to be a rich source of new chemistry: examples include a group of 6-deoxyglycosidic elaiophylin derivatives, including two new antibiotics 11′,12′-dehydroelaiophylin (**3**) and 11,11′-*O*-dimethyl-14′-deethyl-14′-methylelaiophylin (**4**), which were obtained from the culture broth of a marine-derived *Streptomyces* sp. 7-145, along with four known analogues (**5**–**8**). It is worth noting that some of them displayed inhibitory activity against several methicillin-resistant *S. aureus* (MRSA) strains and vancomycin-resistant enterococci (VRE) pathogens, presenting MIC values of 1–2 μg/mL [30]. Chemical investigation of the culture broth of a deep-sea derived *Streptomyces koyangensis* SCSIO 5802 yielded three new polycyclic macrolactones, neoabyssomicins A–C (**9**–**11**), along with two known abyssomicins (**12**,**13**). They were assayed for antibacterial activity and the results showed that only the known abyssomicin **12** could inhibit the growth a panel of Gram-positive pathogens, with MIC values ranging from 3 to 15 μg/mL. However, it was found that neoabyssomicins A and C could inhibit the replication of the HIV-1 virus in MT-2 lymphocytes [31]. Subsequently, a further study on this strain by the same research group led to the discovery of another three new abyssomicin monomers, neoabyssomicins D (**14**), E (**15**), and A_2_ (**16**), together with two dimers neoabyssomicins F (**17**) and G (**18**). Amongst them, the two dimeric compounds displayed inhibitory activity against MRSA strains and vesicular stomatitis virus, respectively [32]. Three 16-membered macrolides, including two known compounds, named dihydrochalcomycin (**19**) and chalcomycin (**20**), in addition to one new compound chalcomycin E (**21**), were discovered from marine-derived *Streptomyces* sp. HK-2006-1. The antibacterial testing result showed that these two known compounds were active in inhibiting *S. aureus*, with MIC values of 32 µg/mL and 4 µg/mL, respectively [33]. Macrolide polyketides were not the only class isolated from *Streptomyces* isolates: another marine-derived *Streptomyces* sp. SCSGAA 0027 afforded four new polyhydroxylated polyketides, namely nahuoic acids B–E (**22**–**25**), which contain a decalin ring. They displayed weak inhibitory activity against the biofilm formation *of Shewanella onedensis* MR-1 [34].

Other actinomycete genera have also been found to produce biologically active compounds. The fermentation broth of marine-derived actinomycete *Micromonospora carbonacea* LS276 provided a new spirotetronate, tetrocarcin Q (**26**), which possessed an unusual 2-deoxy-allose group. The spirotetronate family features an unusual macrolide that is composed of a characteristic tetronic acid, a *trans*-decalin system, together with two sugar side chains. Tetrocarcin Q showed moderate antibacterial activity against *Bacillus subitlis* ATCC 63501, with an MIC value of 12.5 µM [35].

Condensed aromatic polyketides are also well represented in the literature from Chinese researchers, possessing diverse biological activities, including antibacterial, antifungal, anticancer, and antioxidant properties. These include saccharothrixones A–D (**27**–**30**), four new tetracenomycin-type aromatic polyketides, which were isolated from a marine-derived actinomycete *Saccharothrix* sp. 10-10. Among them, saccharothrixone D showed moderate cytotoxicity against the HepG2 cancer cell line in vitro, with an IC_50_ value of 7.5 μM [36]. Stremycins A (**31**) and B (**32**), which are two new angucycline-type antibiotics with antimicrobial activity, were obtained from the culture broth of marine *Streptomyces pratensis* NA-ZhouS1 by induction with high concentrations of nickel ions. They displayed antimicrobial activities against a panel of bacterial strains, including *Bacillus subtillis*, *Pseudomonas aeruginosa*, methicillin resistant *Staphylococcus aureus* (MRSA), *Klebsiella pneumonia*, and *Escherichia coli*, with MIC values ranging from 8 to 16 µg/mL [37]. Chen et al. isolated two new aromatic epimeric polyketides, namely wailupemycins H (**33**) and I (**34**), from the marine-derived *Streptomyces* sp. OUCMDZ-3434 isolated from marine algae, *Enteromorpha prolifera*. These compounds exhibited α-glucosidase inhibitory activity, with K_i_/IC_50_ values of 16.8/19.7 and 6.0/8.3 μM, respectively [38]. Recently, a series of pradimicin-like polyketides, the hexaricins, including five new compounds **35**–**39**, were obtained from a marine sediment that is derived *Streptosporangium* sp. Of these, hexaricins F (**37**) and G (**38**) showed potent antioxidant activity, even stronger than the positive control [39]. Some researchers commonly employ co-culture as a strategy for producing novel chemistry: in a 2018 study, Zhang *et al.* isolated janthinopolyenemycins A (**40**) and B (**41**), which are two novel antifungal polyketides, from the co-culture of two marine-sourced bacteria *Janthinobacterium* spp. ZZ145 and ZZ148. These compounds displayed moderate antifungal activity against *Candida albicans*, with a MIC value of 15.6 μg/mL and a MBC value of 31.25 μg/mL [40].

### 2.2. Fungal Polyketides

Marine fungi are also major producers of diverse polyketides, with varied structures and biological activities. The chemical investigation of extracts from the marine fungus *Dendrodochium* sp. yielded thirteen new 12-membered macrolides, dendrodolides A–M (**42**–**54**). Some of these displayed cytotoxicity against SMMC-7721 and HCT116 cells [41]. Fei He and co-workers also reported macrolides: sumalactones A–D (**55**–**58**), along with two known compounds curvularin (**59**) and dehydrocurvularin (**60**), were isolated from a marine fungus *Penicillium Sumatrense* that was isolated from deep-sea sediments. They possessed a unique 10- or 11-membered macrolide skeleton and dehydrocurvularin displayed potent inhibitory activity against NO production, which is associated with inflammatory disorders [42]. Recently, Wu et al. isolated a group of new polyester polyketides, namely hansforesters A–M (**61**–**73**), from a sponge-derived fungus *Hansfordia sinuosae*, of which hansforester A displayed strong inhibitory activity against a wide spectrum of bacterial strains, with MIC values of 3.9 to 15.6 µM [43].

However, aromatic polyketides with diverse biological activities are much more commonly reported from marine-derived fungi. For example, Zhang et al. identified four new tetralone derivatives, including heterodimeric clindanones A (**74**) and B (**75**) and homodimeric cladosporols F (**76**) and G (**77**) from a deep-sea isolate of the fungus *Cladosporium cladosporioides* HDN14-342. Cladosporol G displayed good cytotoxicity against HeLa cells, with an IC_50_ value of 3.9 µM [44]. Eight antifouling and antibacterial polyketides (**78**–**85**), including two previously unreported examples, 6,8,5′,6′-tetrahydroxy-30-methylflavone (**78**) and paecilin C (**79**), were isolated from the gorgonian coral-associated fungus *Penicillium* sp. SCSGAF 0023. Compounds **78** and **83**–**85** displayed antifouling activity against larvae settlement of *Balanus amphitrite*, with EC_50_ values of 6.7–17.9 µg/mL, while compounds **80**–**82** showed antibacterial activity against four bacterial strains [45]. A study on the fermentation broth of the sponge-derived fungus *Aspergillus* sp. F40 afforded two new polyketides, versiconol B (**86**) and oxisterigmatocystin I (**87**), the former compound displayed weak antimicrobial activity against *S. aureus* and *V. parahaemolyticus* [46]. Liu et al. reported the isolations of several novel anti-inflammatory polyketides, including a pair of enantiomers (+)- and (−)-ascomindone D (**88**,**89**), which incorporate a novel 2,3-diaryl indone moiety, and two new prenylated polyketides ascomfurans C (**90**) and ascomarugosin A (**91**), from the mangrove-derived fungus *Ascomycota* sp. SK2YWS-L. The enantiomers displayed anti-inflammatory activities, while also inhibiting the production of NO with IC_50_ values of 17.0 and 17.1 µM, respectively [47]. Polyketides that were sourced from marine fungi have also proved useful in research on immunosuppressive agents. In a 2016 report, a group of polyketides (**92**–**100**), including two new benzophenone derivatives, peniphenone (**92**), and methyl peniphenone (**93**), were obtained from the mangrove endophytic fungus *Penicillium* sp. ZJ-SY2. These compounds showed significant immunosuppressive activity, with IC_50_ values ranging from 5.9 to 9.3 µg/mL [48].

Many of these compounds are variations on known structure classes; however, a few highly unusual structures have been reported over the past decade. Zhu et al. isolated a new cyctotoxic hybrid polyketide, cladodionen (**101**) from a marine fungus *Cladosporium* sp. OUCMDZ-1635. It existed as a mixture of two geometric isomers and exhibited significant cytotoxicity to several cancer cells lines, with IC_50_ values between 9–19 µM [49]. Many of the fungal polyketides that were reported are citrinin derivatives: a novel antimitotic polyketide dicitrinone D (**102**) was discovered by Chen et al. from a marine sediment-derived fungus *Penicillium citrinum*. It showed cytotoxic activity against many tumor cells, especially the SPC-A1 cells, while not displaying significant toxicity against normal cells: several assays suggested that it was a microtubule-destabilizing agent [50]. Chemical investigation of the deep-sea-sourced *Aspergillus* sp. 16-02-1 afforded nine new C_9_ polyketides (**103**–**111**), including aspiketolactonol, aspilactonols A–F, and epiaspinonediol, along with five known antibiotics. They showed inhibitory activities against a group of cancer cell lines, including K562, HL-60, HeLa, and BGC-823 [51]. Another marine mangrove-derived fungus *Aspergillus ochraceus* MA-15 produced three new polyketides, asperochrins A–C (**112**–**114**). Among them, asperochrins A was the most effective, and it showed selective antibacterial activity against aquatic pathogenic bacteria *Aeromonas hydrophila*, *Vibrio anguillarum*, and *Vibrio harveyi*, with IC_50_ values ranging from 0.5 to 64.0 µg/mL [52]. Fang et al. isolated a new cyclopentenone, named 5-hydroxycyclopenicillone (**115**), from a sponge-derived fungus *Trichoderma* sp. HPQJ-34. Intriguingly, it displayed moderate anti-oxidative, anti-Aβ fibrillization properties, and neuroprotective effects. Overall, Chinese isolates of marine fungi have proved to be a rich source of new polyketides [53].

## 3. Peptides

### 3.1. Bacterial Peptides

**Figure 4:**
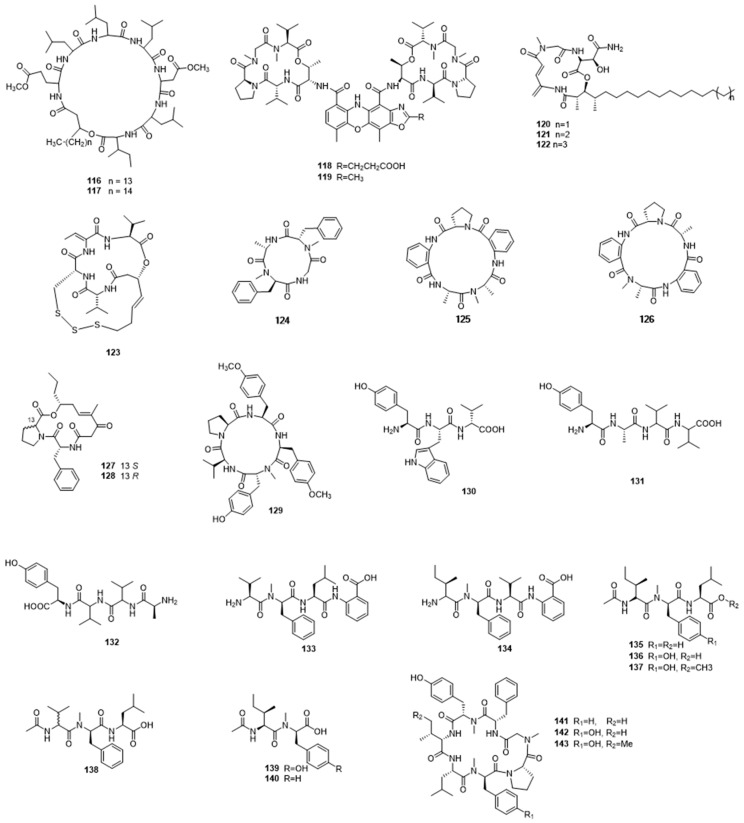
Peptides **116**–**143** from marine microbes.

Marine bacteria could produce diverse peptides, which are a group of compounds that are composed of amino acids (Figure 4). We have included in this class all compounds that are primarily amino-acid derived, which are not alkaloids. For example, Xiu et al. isolated two pumilacidin-like cyclic lipopeptides (**116**,**117**) from the marine-derived bacterium *Bacillus* sp. 176. Intriguingly, they showed potent motility suppression activity against marine bacterium *Vibrio alginolyticus* [54]. Many of the peptide compounds that were derived from marine bacteria displayed remarkable anticancer and antibacterial activity. Neo-actinomycins A and B (**118**,**119**), which are two new members of a group of well-known chromopeptides, were obtained from the marine-derived *Streptomyces* sp. IMB094. Neo-actinomycin A displayed nanomolar cytotoxic activities against human cancer HCT116 and A549 cell lines, with IC_50_ values of 38.7 and 65.8 nM, respectively. In addition, it also exhibited moderate inhibitory activities against methicillin-resistant *Staphylococcus aureus* (MRSA) and vancomycin-resistant Enterococci (VRE) strains [55]. Marine-derived *Micromonospora chalcea* FIM 02-523 afforded three new cyclic depsipeptides, rakicidins G–I (**120**–**122**). They were composed of four unusual amino acids, with a long aliphatic chain attaching to the peptide ring. They displayed potent antitumor activities against HCT-8 and PANC-1 cancer cell lines under hypoxic conditions and also showed antibacterial activities against a panel of bacteria, with MIC values ranging from 0.125–8 µg/mL. Amongst them, rakicidin G was the most effective and it showed very strong antibacterial activity against *Peptostreptococcus anaerobius* ATCC 27337, with an MIC of less than 0.125 mg/mL [56]. Zhou et al. identified a highly cytotoxic depsipeptide, chromopeptide A (**123**), from the marine sediment-derived bacterium *Chromobacterium* sp. HS-13-94, which exhibited inhibitory activity against HL-60, K-562, and Ramos cells, with IC_50_ values of 7.7, 7.0, and 16.5 nmol/L, respectively [57].

### 3.2. Fungal Peptides

Marine fungi are also a major resource of biological peptides. Three new cyclopeptides, versicotides D–F (**124**–**126**) were isolated from the gorgonian-derived fungus *Aspergillus versicolor* LZD-14-1. Versicotides E and F possessed very rare structures that have two anthranilic acid residues and a proline in a single cyclic peptide system. They showed significant lipid-lowering activities by regulating cholesterol efflux and influx [58]. Chemical investigation on the culture extracts of mangrove-derived fungus *Sarocladium kiliense* HDN11-112 led to the discovery of two new cyclic depsipeptides saroclides A (**127**) and B (**128**). These did not show any inhibitory activity against a series of tested cancer cells and bacterial strains, but saroclide A displayed some lipid-lowering activities. Marine fungi from Chinese waters have also been shown to produce antiviral peptides [59]. In 2017, Ma et al. isolated four new peptides, including one new cyclic pentapeptide aspergillipeptide D (**129**) and three linear peptides aspergillipeptides E–G (**130**–**132**) from the marine gorgonian-derived fungus *Aspergillus* sp. SCSIO 41501, by increasing the amount of L-tryptophan in basic culture medium. Aspergillipeptides D and F exhibited potent antiviral activity against herpes simplex virus type 1 (HSV-1), with IC_50_ values of 9.5 and 19.8 µM, respectively [60]. The marine-derived fungus *Simplicillium obclavatum* EIODSF 020 that was isolated from deep-sea sediment produced a series of new linear peptides, namely simplicilliumtides A–H (**133**–**140**). Among them, simplicilliumtide D showed potent antifouling activity against *Bugula neritina* larvae settlement [61]. In a 2012 report, Chen et al. described three new cycloheptapeptides, cordyheptapeptides C–E (**141**–**143**), from the marine-derived fungus *Acremonium persicinum* SCSIO115. Cordyheptapeptides C and E showed cytotoxic activities against SF-268, MCF-7, and NCI-H460 cancer cell lines, with IC_50_ values that ranged from 2.5 to 12.1 μM [62].

## 4. Alkaloids

### 4.1. Bacterial Alkaloids

**Figure 5:**
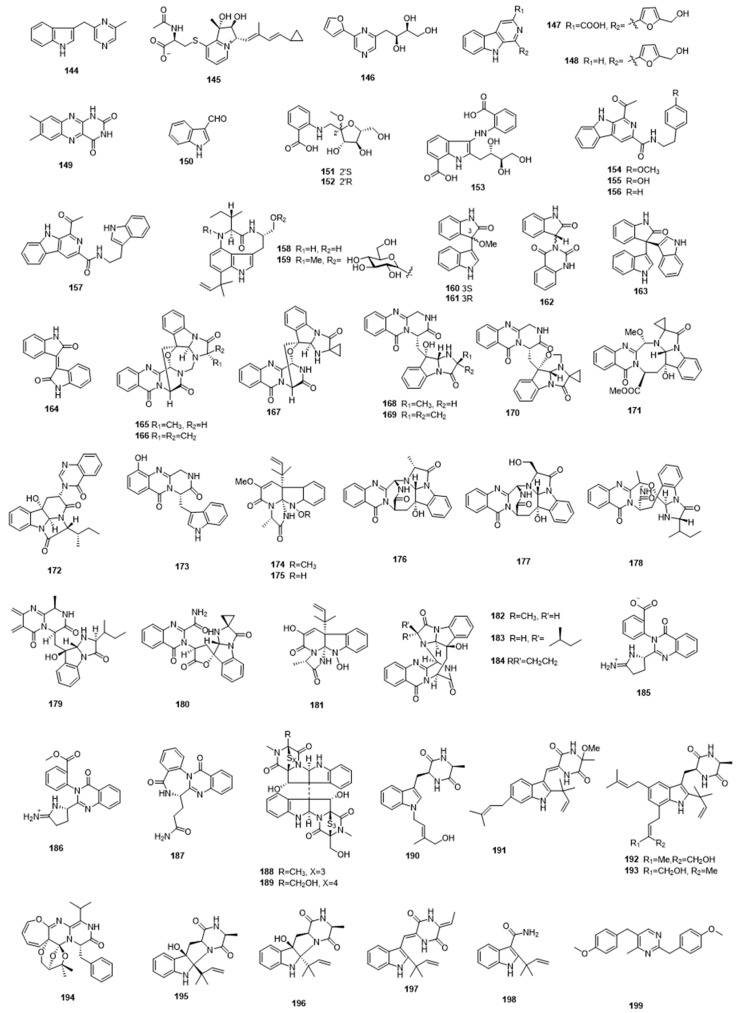
Alkaloids **144**-**199** from marine microbes.

**Figure 6:**
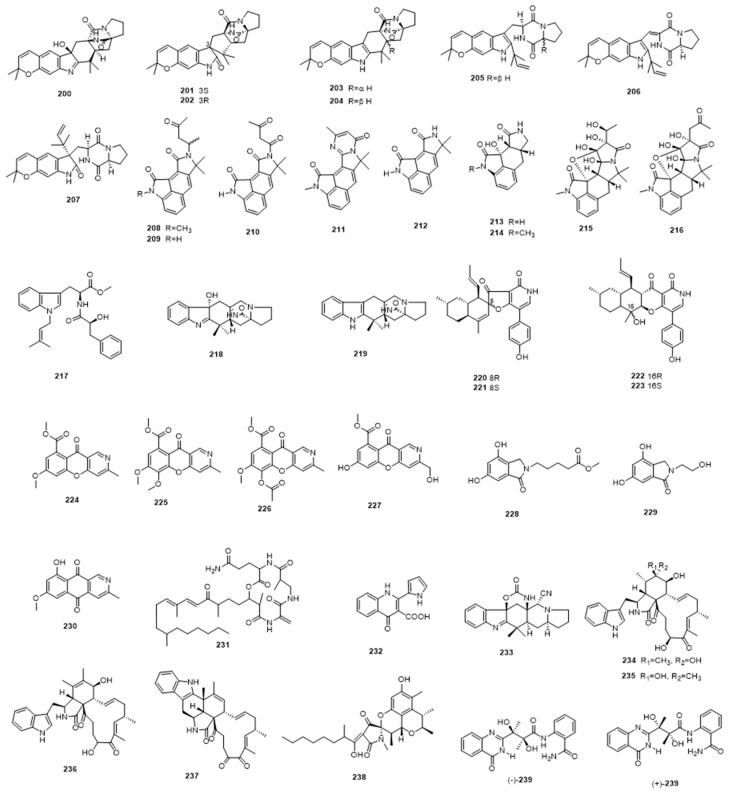
Alkaloids **200**–**239** isolated from marine-derived microbes.

Alkaloids that are produced by marine bacteria and fungi are a large group of compounds with diverse structures and bioactivities (Figure 5; Figure 6). Amongst bacteria, alkaloids were largely reported from members of the actinomycete genus, including some novel species. For example, the chemical investigation of culture extract from a novel deep-sea actinomycete *Serinicoccus profundi* sp. nov. belonging to the family *Intrasporangiaceae*, suborder *Micrococcineae,* led to the discovery of 3-((6-methylpyrazin-2-yl) methyl)-1*H*-indole (**144**), a previously unreported alkaloid, which showed weak antibacterial activity against *Staphylococcus aureus* [63]. Another antibacterial alkaloid, streptopertusacin A (**145**), which is a unique indolizinium alkaloid, was obtained from the marine-derived *Streptomyces* sp. HZP-2216E, and it displayed moderate antibacterial activity against methicillin-resistant *Staphylococcus aureus* (MRSA) [64]. In addition to antibacterial actives, alkaloids with antiviral activity were also obtained from marine actinomycetes. In 2013, Wang et al. isolated a new alkaloid, 2-(furan-2-yl)-6-(2*S*,3*S*,4-trihydroxybutyl) pyrazine (**146**), along with twelve known compounds, from the mangrove-derived actinomycete *Jishengella endophytica* 161111. Four of these known compounds (**147**–**150**) showed antiviral activity against the influenza A virus subtype H1N1 [65]. Recently, Che et al. isolated three new anthranilate-containing alkaloids, the anthranosides A–C (**151**–**153**), from the marine sponge-derived actinomycete *Streptomyces* sp. CMN-62. Among them, anthranoside C displayed antiviral activity against the influenza A H1N1 virus, with an IC_50_ value of 171 μM [66]. Another rare marine actinomycete *Marinactinospora thermotolerans* SCSIO 00652, belonging to the family *Nocardiopsaceae,* afforded six new compounds, including four new β-carboline alkaloids (**154**–**157**) and two new indolactam alkaloids (**158**,**159**). They showed significant antiplasmodial activities against *Plasmodium falciparum* lines 3D7 and Dd2, with IC_50_ values ranging from 1.92 to 36.03 μM [67]. Three indole-based alkaloids, shewanellines A–C (**160**–**162**), together with twelve known compounds, were discovered from the deep-sea bacterium *Shewanella piezotolerans*. Shewanellines C and two of the known compounds (**163**,**164**) showed potent antitumor activity against the HL-60 cell line [68]. As can be seen from above, although the amount of alkaloids derived from bacteria is not extensive, they exhibit a wide variety of activities, including antibacterial, antiviral, antiplasmodial, and anticancer properties.

### 4.2. Fungal Alkaloids

However, marine fungi were the most prolific source for the production of various alkaloids over the time period surveyed. Quinazoline-derived alkaloids are one of most commonly seen classes of alkaloids. In 2017, Cheng et al. reported the isolation of eleven new fumiquinazoline-type alkaloids, unique polycyclic natural products with diverse scaffolds, namely the versiquinazolines A–K (**165**–**175**), from the gorgonian-derived fungus *Aspergillus versicolor* LZD-14-1. Some of them displayed inhibitory activities against thioredoxin reductase, with IC_50_ values ranging from 12 to 20 μM [69]. Subsequently, additional chemical investigation on this microbial strain by the same research group led to the discovery of another six new fumiquinazoline-type alkaloids, versiquinazolines L−Q (**176**–**181**), among which versiquinazolines P and Q also showed inhibitory activity against thioredoxin reductase, with IC_50_ values of 13.6 μM and 12.2 μM, respectively [70]. Another fungus, *Aspergillus versicolor* LCJ-5-4, isolated from a marine coral, was reported to produce three new tryptophan-derived quinazolinone alkaloids, cottoquinazolines B–D (**182**–**184**). Among them, Cottoquinazoline D was a unique natural product that possessed a 1-aminocyclopropane-1-carboxylic acid residue; it showed moderate inhibitory activity against *Candida albicans* [71]. Three new anticancer alkaloids, the auranomides A–C (**185**–**187)**, were discovered from the marine-derived fungus *Penicillium aurantiogriseum*. Auranomides A and B possessed a new scaffold that included a quinazolin-4-one that was coupled with pyrrolidin-2-iminium moiety. They displayed significant cytotoxic activity against human tumor cells, with auranomide B displaying nanomolar activity against HEPG2 cells, presenting an IC_50_ value of 0.097 μM/mL [72].

Piperazine-derived alkaloids, especially diketopiperazines, are another common alkaloid class discovered from marine fungi. Two new cyctotoxic epipolythiodioxopiperazines alkaloids that possessed a bridged polysulfide piperazine ring, namely chetracins E (**188**) and F (**189**), were discovered from the marine-derived fungus *Acrostalagmus luteoalbus* HDN13-530 by Yu et al. They exhibited remarkable cytotoxicity against a wide spectrum of cancer lines, with IC_50_ values ranging from 0.2 to 3.6 μM [73]. Algae-derived fungi are also an excellent source of alkaloids. Chemical investigation of the culture extract of a marine algal-derived endophytic fungus *Eurotium cristatum* led to the isolations of thirteen indolediketopiperazine alkaloids, as exemplified by compounds **190**–**193**. The new compound **191** displayed cytotoxic properties in a brine shrimp assay [74]. Zhang et al. reported a new 3H-oxepine-containing diketopiperazine-type alkaloid, namely varioxepine A (**194**), from the marine algal-derived fungus *Paecilomyces variotii.* It showed inhibitory activity against the plant pathogenic fungus *Fusarium graminearum* [75]. Five new alkaloids, including three new prenylated indole 2,5-diketopiperazine alkaloids (**195**–**197**), one new indole alkaloid (**198**), and one new *bis*-benzyl pyrimidine derivative (**199**), the eurotiumins A–E, along with nine known analogues, were obtained from the marine-derived fungus *Eurotium* sp. SCSIO F452. They showed significant radical scavenging activities in a DPPH assay [76]. Marine fungi also produce indole-derived alkaloids. Recently, Li et al. isolated eight rare linearly-fused prenylated indole alkaloids that contained a pyrano[3,2-f]indole unit, named asperversiamides A–H (**200**–**207**), from the marine-derived fungus *Aspergillus versicolor*. Asperversiamide J showed a significant inhibitory activity against NO production by inhibiting inducible nitric oxide synthase (iNOS), with an IC_50_ value of 5.39 μM [77]. Xu et al. reported the isolations of a group of oxindole alkaloids, including nine new compounds, namely cyclopiamides B–J (**208**–**216**), from the marine fungus *Penicillium commune* DFFSCS026, which was isolated from a deep-sea sediment; several of these showed moderate brine shrimp lethality [78]. In a 2018 report, Zhou et al. isolated a new indole alkaloid, misszrtine A (**217**), from the marine sponge-derived fungus *Aspergillus* sp. SCSIO XWS03F03. Misszrtine A displayed remarkable inhibitory activity against HL60 and LNCaP cell lines, with IC_50_ values of 3.1 µM and 4.9 µM, respectively [79]. Mutant strains that were obtained from mutation of marine microbes have also provided an abundance of natural products. In 2017, Chen et al. discovered two prenylated indole alkaloids, named penicimutamides D and E (**218**,**219**), from one mutant strain of a marine-derived *Penicillium purpurogenum* G59 via diethyl sulfate (DES) mutagenesis. The compounds showed weak inhibitory activity against the tested cancer cell lines [80].

Some other types of alkaloids have also be obtained from marine fungi. Campyridones A–D (**220**–**223**), four new pyridone alkaloids, together with a known ilicicolin, were reported from a mangrove endophytic fungus *Campylocarpon* sp. HDN13-307 by Li and co-workers. Campyridone D displayed cytotoxicity against HeLa cells, with IC_50_ values of 8.8 and 4.7 μM, respectively [81]. The mangrove endophytic fungus *Diaporthe phaseolorum* SKS019 afforded six new alkaloids, which included four new chromeno[3,2-c]pyridines (**224**–**227**) and two new isoindolinones (**228**,**229**), along with three known compounds. Among them, the known compounds **230**, **231** showed potent anticancer activity against MDA-MB-435, HCT116, and NCI-H460 human cancer cell lines [82]. Shao et al. isolated a new pyrrolyl 4-quinolinone alkaloid that possessed an unprecedented ring system, namely penicinoline (**232**), from a mangrove endophytic fungus *Penicillium* sp. It displayed inhibitory activity against 95-D and HepG2 cell lines, with IC_50_ values of 0.57 and 6.5 µg/mL, respectively [83].

Marine microbes also produce some truly unusual alkaloid compounds. Aspeverin A (**233**), which is a novel carbamate- and cyano-containing alkaloid, was reported from the marine fungus *Aspergillus versicolor* strain that was isolated from a green alga. It showed potent inhibitory activity against phytoplankton (*Heterosigma akashiwo*), with EC_50_ values of 6.3 and 3.4 μg/mL after 24 and 96 h, respectively [84]. A study regarding the chemical constituents of a culture extract from the marine fungus *Chaetomium globosum* led to the discovery of two new cytochalasan alkaloids, namely cytoglobosins H (**234**) and I (**235**), along with seven analogues. Among them, chaetoglobosin E (**236**) exhibited strong antiproliferative activity against LNCaP and B16F10 cell lines, with IC_50_ values of 0.62 and 2.78 µM, respectively [85]. Subsequently, in a 2017 report, Zhu et al. discovered another new chaetoglobosin, penochalasin K (**237**), which contained a fused ring system, from the mangrove endophytic fungus *Penicillium chrysogenum* V11. It showed moderate antimicrobial activities against *Colletotrichum gloeosporioides* and *Rhizoctonia solani*, with MIC values of 6.13 and 12.26 μM, respectively, in addition to moderate activity against a panel of cancer cell lines [86]. Another potent antitumor alkaloid agent, penicitrinine A (**238**), which possessed a unique spiro skeleton, was discovered from the marine fungus *Penicillium citrinum*. It showed inhibitory activities against multiple tumor types, especially on human malignant melanoma cell A-375 [87]. In a 2014 report, He et al. obtained a new unusual alkaloid, named penicilliumine (**239**), from the marine-derived fungus *Penicillium commune* 366606. It existed as a racemic mixture of (−)-penicilliumine and (+)-penicilliumine, and showed inhibitory activity against acetylcholinesterase [88].

## 5. Terpenoids, Terpenes and Meroterpenoids

**Figure 7:**
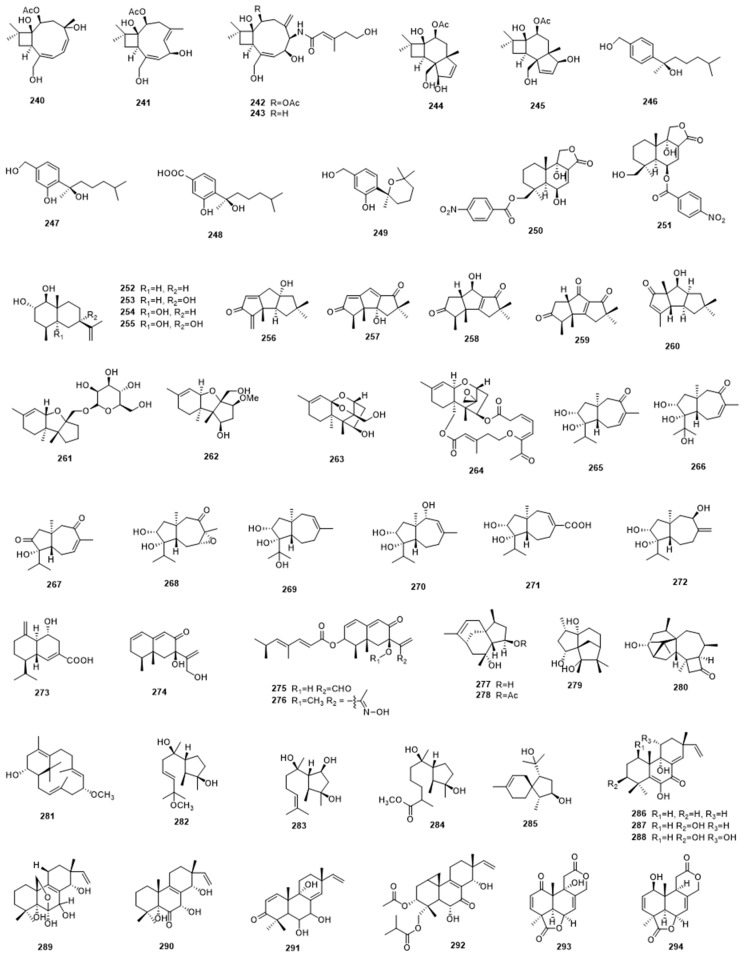
Terpenoids **240**–**294** from marine-derived microbes.

**Figure 8:**
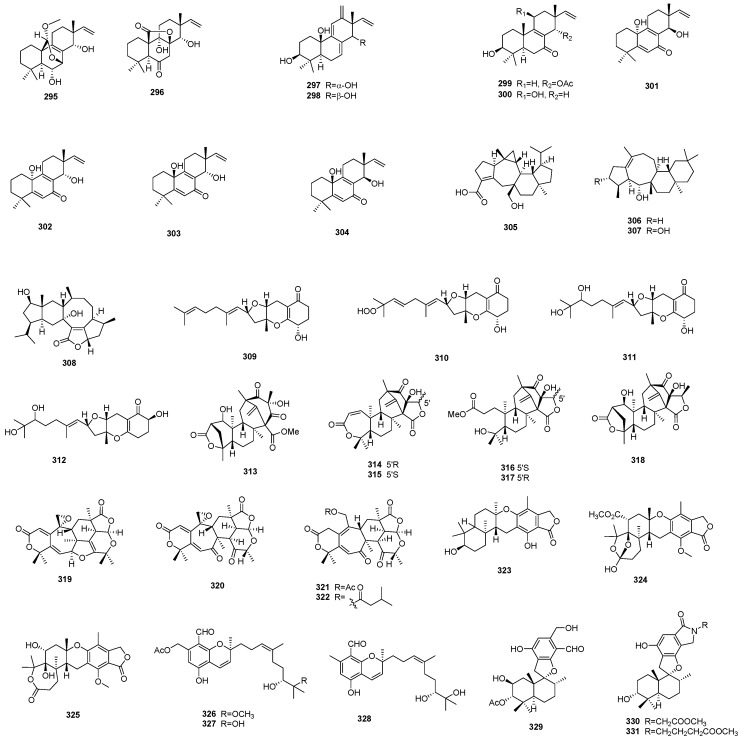
Terpenoids **295**–**331** from marine-derived microbes.

Marine fungi could also produce a variety of structurally different terpenoid compounds with interesting biological activities, such as eremophilane-type and carotene-type sesquiterpene, bisabolane-type sesquiterpenoids, eudesmane-type sesquiterpenoids, pimarane-type diterpenes, and other types, which have been widely reported by Chinese researchers (Figure 7 and Figure 8). Amongst them, multiple types of sesquiterpenoid or sesquiterpene compounds are most commonly observed.

Chemical investigation on the culture extract of sponge-associated fungus *Hansfordia sinuosae* afforded six new caryophyllene-type sesquiterpenoids that possess a unique *bicyclo*[2.7.0]undecane skeleton, named punctaporonins H–M (**240**–**245**). Among them, punctaporonins K showed potent lipid-lowering activity that could reduce the intracellular levels of triglycerides and total cholesterol [89]. Another sponge-derived fungus *Aspergillus* sp. afforded four new antibacterial bisabolane-type sesquiterpenoids (**246**–**249**). They showed either broad spectrum or selective antibacterial activity against the eight tested bacterial strains [90]. Some sesquiterpenoids also exhibit significant anticancer activities. Fang et al. isolated a highly unusual nitrobenzoyl sesquiterpenoid, 6b,9a-dihydroxy-14-*p*-nitrobenzoylcinnamolide (**250**), together with a known analogue, insulicolide A (**251**), from the marine-derived fungus *Aspergillus ochraceus* Jcma1F17. These both displayed significant anticancer activity against a panel of cancer cell lines, while **251** also displayed antiviral activities against H3N2 and EV71 [91]. The mangrove-derived endophytic fungus *Penicillium* sp. J-54 afforded four new eudesmane-type sesquiterpenoids, namely penicieudesmol A–D (**252**–**255**). Among them, penicieudesmol B displayed weak cytotoxicity against K-562 cells [92]. Li et al. isolated five new triquinane-type sesquiterpenoids, namely chondrosterins A–E (**256**–**260**), from the soft coral-associated fungus *Chondrostereum* sp. Chondrosterin A showed low micromolar cytotoxic activities against the cancer lines A549, CNE2, and LoVo [93].

Chromatographic separation of the culture extract from the sponge-associated fungus *Stachybotrys chartarum* led to the isolations of a group of cytotoxic trichothecene-type sesquiterpenes, including four previously unreported ones, namely chartarenes A–D (**261**–**264**). They exhibited potent antitumor activity against a panel of tumor cell lines, including HCT-116 and HepG2, with IC_50_ values < 10 μg/mL, in addition to inhibitory activity against tumor growth-related tyrosine kinases, including FGFR3, IGF1R, PDGFRb, and TRKB [94]. Chemical investigation of the marine fungus *Trichoderma virens*, which was isolated from marine red alga, led to the discovery of eight new carotane sesquiterpenes, named trichocarotins A–H (**265**–**272**), and one new cadinane sesquiterpene, named trichocadinin A (**273**), some of which showed inhibitory activity against the growth of some marine plankton species [95]. Dendryphiellins H–J (**274**–**276**), three new eremophilane sesquiterpenes, were isolated from the marine-derived fungus *Cochliobolus lunatus* SCSIO41401. Dendryphiellin I displayed significant cytotoxicities against five cancer cell lines, with an IC_50_ value of 1.4 to 4.3 μM, in addition to antibacterial activities against bacterial species, with MIC values of 1.5 to 13 μg/mL, respectively. The unusual aldoximine derivative dendryphiellin J also showed potent cytotoxicities against ACHN and HepG-2 cells, with IC_50_ values of 3.1 and 5.9 μM, respectively [96]. The marine-derived fungus *Penicillium bilaiae* MA-267 provided two novel sesquiterpenes (**277**,**278**) that possessed a *tricyclo*[6.3.1.0^1,5^]dodecane skeleton, they showed activity against the plant pathogenic fungus *Colletotrichum gloeosporioides* [97]. While the majority of terpenes from marine-derived microbes come from fungi, a few have been reported from bacteria. One example is a new caged sesquiterpene, named strepsesquitriol (**279**), which was isolated from the marine-derived actinomycete *Streptomyces* sp. SCSIO 10355, which showed moderate inhibitory activity against lipopolysaccharide-stimulated tumor necrosis factor (TNFα) production in RAW264.7 macrophages [98].

In addition to sesquiterpenes, diterpenes were also obtained from marine fungi in many instances. In 2018, Song et al. isolated six new terpenes, including one harziane diterpene (**280**), one proharziane diterpene (**281**), three cyclonerane sesquiterpene (**282**–**284**), and one acrorane sesquiterpene (**285**), from the marine fungus *Trichoderma harzianum X-5* isolated from a marine brown alga. They exhibited inhibitory activity against some marine phytoplankton species [99]. Pimarane diterpenes are a widely distributed class of terpenes that are produced by a wide variety of organisms, and marine-derived fungi are no exception [100]. In 2012, Sun et al. reported the isolations of five new oxygenated pimarane diterpenes, named scopararanes C–G (**286**–**290**), from a marine sediment-derived fungus *Eutypella scoparia* FS26. Scopararanes C and D showed moderate cytotoxic activities against the MCF-7 tumor cell line [101]. Later, another two new pimarane-type diterpenes, scopararanes H and I (**291**,**292**), were isolated from the same research group as the marine sediment-derived fungus *Eutypella* sp. FS46. Scopararane I showed moderate inhibitory activities against the tested tumor cell lines [102]. Another sediment-derived fungus, *Aspergillus wentii* SD-310, provided a wealth of new diterpenoid compounds, including two new tetranorlabdane diterpenoids, named asperolides D (**293**) and E (**294**), six new isopimarane-type diterpenoid derivatives, named wentinoids A–F (**295**–**300**), and four new uncommon 20-nor-isopimarane diterpenoid epimers, aspewentins I–L (**301**–**304**). Some of them exhibited promising selective anticancer or antimicrobial activity [103,104,105].

*Aspergillus* species have been shown to be a particularly rich source of terpenoid compounds, including sesterterpenoids: Asperterpenoid A (**305**), a novel pentacyclic sesterterpenoid, was discovered from a mangrove endophytic fungus *Aspergillus* sp. 16-5c. It showed strong inhibitory activity against *Mycobacterium tuberculosis* protein tyrosine phosphatase B (mPTPB), with an IC_50_ value of 2.2 μM [106]. Later, the mangrove endophytic fungus *Aspergillus* sp. 085242 afforded another two similar sesterterpenoids with an unusual 5/8/6/6 tetracyclic ring skeleton, which were named asperterpenols A (**306**) and B (**307**). In contrast to asperterpenoid A, they showed potent acetylcholinesterase (AChE) inhibitory activity, with IC_50_ values of 2.3 and 3.0 μM, respectively [107].

Meroterpenes and meroterpenoids are also commonly seen compounds that are produced by marine microbes. They are hybrid natural products assembled by terpene or terpenoids moieties with other precursors, such as polyketide [108], and they display a diverse range of useful biological activities, as exemplified below. Chemical investigation of the culture extract from the marine fungus *Alternaria* alternata k21-1 isolated from a marine alga afforded a new sesterterpene, named sesteralterin (**308**), and four new meroterpenes, named tricycloalterfurenes A–D (**309**–**312**). Some of them showed selective inhibitory activity against phytoplankton [109]. Brasilianoids A–F (**313**–**318**), six new meroterpenoids, were reported from the sponge-associated fungus *Penicillium brasilianum* in 2018. Brasilianoid A could significantly stimulate the expression of filaggrin and caspase-14 in HaCaT cells in a dose-dependent manner, while brasilianoids B and C showed moderate inhibitory activity against NO production in LPS-induced RAW 264.7 macrophages [110]. Chen et al. isolated four anti-inflammatory meroterpenoids, named amestolkolides A–D (**319**–**322**), from the mangrove endophytic fungus *Talaromyces amestolkiae* YX1. Among them, amestolkolides B showed anti-inflammatory activity against LPS-activated NO production in RAW264.7 cells in vitro, with IC_50_ value of 1.6 μM [111]. Meroterpenoids have also shown good activity in antiviral assays: the sponge-derived fungus *Aspergillus aureolatus* HDN14-107 generated three new meroterpenoids, named austalides S-U (**323**–**325**). Austalides U displayed inhibitory activity against influenza virus A (H1N1) [112]. In 2017, Zhang et al. isolated six new meroterpenoids, named stachybonoids A–F (**326**–**331**), together with three known phenylspirodrimanes, from the crinoid-derived fungus *Stachybotrys chartarum* 952. Among them, stachybonoid A showed inhibitory activity against the replication of dengue virus, while stachybonoid F showed anti-inflammatory activity against LPS-activated NO production in RAW264.7 cells [113].

## 6. Mixed-Structure Compounds

**Figure 9:**
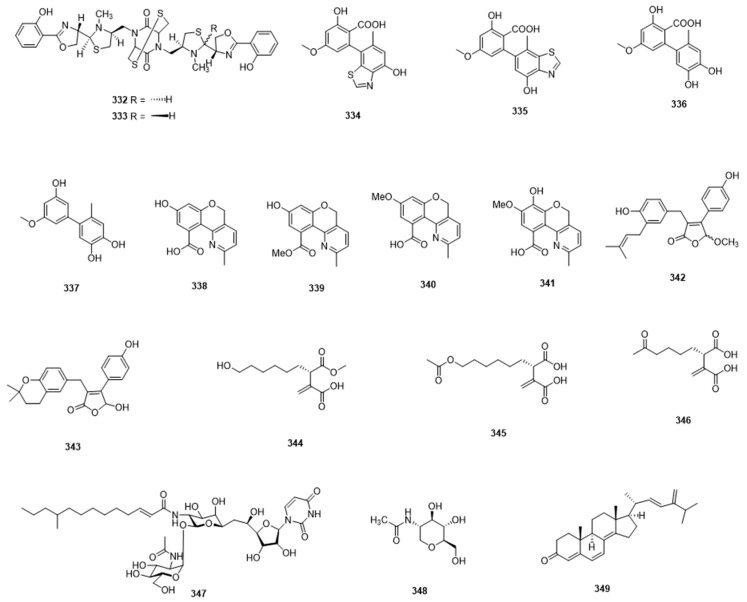
Mixed biosynthesis and miscellaneous compounds **332**–**349**.

Many of the secondary metabolites from marine-derived microbes are structurally hybrid compounds (Figure 9), similar to the meroterpenoids mentioned earlier. In 2013, Liu et al. isolated two new oxazole-thiazole siderophores, namely tetroazolemycins A (**332**) and B (**333**), from a *Streptomyces olivaceus* strain, isolated from deep-sea water. They showed an affinity for a range of cations, including Fe^3+^, Cu^2+^, and Zn^2+^ [114]. Chemical investigation of culture extract of a sponge-derived fungus *Alternaria* sp. afforded two new altenusin-thiazole hybrids, named altenusinoides A (**334**) and B (**335**), along with three known compounds. Amongst them, the known compounds **336**–**337** showed DPPH free-radical-scavenging activities; **337** also showed COX-2 inhibitory activity [115]. Chen et al. identified four new chromenopyridine derivatives, phochrodines A–D (**338**–**341**), from the mangrove entophytic fungus *Phomopsis* sp. 33#. Phochrodines B and D showed inhibitory activity against NO production, while phochrodine D also exhibited DPPH free-radical-scavenging activities [116].

## 7. Other Compounds

Marine microbes have also been shown to produce compounds that cannot easily be assigned to a particular structure class; these are briefly discussed here. In a 2018 report, Sun et al. isolated a group of butenolide derivatives, including two new compounds (**342**,**343**) from a marine sponge-derived fungus *Aspergillus terreus*. They displayed potent inhibitory activities against α-glucosidase [117]. Chemical investigation of culture extract of another marine fungus, *Aspergillus niger*, led to the discovery of three new itaconic acid derivatives, namely asperitaconic acids A–C (**344**–**346**). They showed inhibitory activity against *Staphylococcus aureus*, with MIC values of 16–32 μg/mL [118]. Tunicamycin E (**347**), a new nucleoside antibiotic, along with six analogues, was obtained from the marine-derived *Streptomyces xinghaiensis* SCSIO S15077. They displayed moderate antifungal activity against two *Candida albicans* strains, and very strong inhibitory activity against *Bacillus thuringiensis*, with MIC values of 0.008–2 μg/mL [119]. Huang et al. obtained one 1-deoxy-*N*-acetylglucosamine compound (**348**) from a marine bacterium *Virgibacillus dokdonensis* MCCC 1A00493 isolated from deep-sea polymetallic nodules. It displayed significant inhibitory activity against *Xanthomonas oryzae* pv. *oryzae*, which is a pathogenic bacteria causing rice bacterial blight [120]. In a few instances, the reports describe the isolation of new compounds from multiple structure classes: Chen et al. isolated six steroid derivatives, including a new one (**349**), together with five butyrolactone derivatives, from a gorgonian-derived fungus *Aspergillus* sp. Some of them showed antifouling activity against larval settlement of the barnacle *Balanus amphitrite* and antibacterial activity against *Staphylococcus aureus* [121].

## 8. Discussion

**Figure 10:**
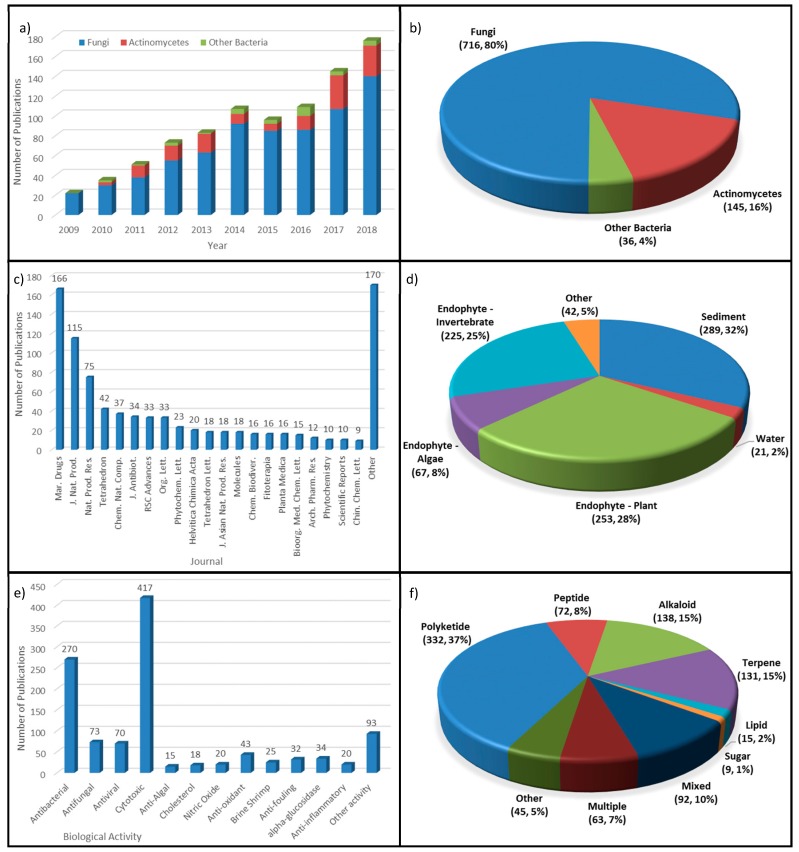
Analysis of publications analyzed in this review. (**a**) Number of publications each year, split my organism type; (**b**) percentage breakdown of publications by organism type; (**c**) publications by journal; (**d**) percentage of publications by sample collection location; (**e**) number of publications for each biological activity; and, (**f**) percentage of publications by structure class of new/active compounds.

A total of 897 papers describing the isolation of biologically active compounds from marine-derived microbes were analyzed for this review (Supplementary Table S1). One of the most notable and immediately apparent trends is the dramatic increase in the number of China-based publications focusing on the chemistry of marine microbes over the past decade. A mere 22 papers on the topic were published in English-language journals in 2009, which has increased more than sevenfold over the past decade, with 176 papers being published in 2018 (Figure 10a). This increase may be due to increasing numbers of groups conducting this research, or it might represent a shift from publication in local Chinese journals to international journals. The overwhelming focus on fungi is also clear, with 80% of all reports describing the isolation of bioactive compounds from fungi, 16% from actinomycetes, and only 4% from other bacteria (Figure 10b); there does not appear to be any consistent change in this composition over the 10 years that were monitored for this review.

The major venue for publication has been *Marine Drugs* (166 publications, 18.5%), followed by the *Journal of Natural Products* (115 publications, 12.8%) and *Natural Product Research* (75, 8.4%) (Figure 10c). Given the subject material, the popularity of the former is very understandable, while the latter two also strongly featured in a 2018 review on China-based natural products research from all sources [15]. Other popular journals included *Tetrahedron* (42, 4.7%), *Chemistry of Natural Compounds* (37, 4.1%), the *Journal of Antibiotics* (34, 3.8%), *Organic Letters* (33, 3.7%), and RSC *Advances* (33, 3.7%). 

Marine sediments were the most popular location for the collection of microbial strains (289 papers, 32%), though endophytic microbes from plants (253, 28%), invertebrates (225, 25%), and algae (67, 7.4%) were also popular (Figure 10d). While marine algae and invertebrates are a common source of microbial endophytes in many countries, marine plants are not so. Their popularity in China can be largely attributed to the presence of several research groups focusing on the chemistry of mangrove endophytes, in particular, fungi, which can be a rich source of novel chemistry [23,122,123].

In terms of structure class, examples of the various structural classes has been discussed in depth above, but it was clear that polyketides made up the largest proportion of new and active compounds reported (332, 37%), distantly followed by alkaloids (138, 15%) and terpenes (131, 15%) (Figure 10e); compounds with mixed biosynthetic origin (92, 10%) were also well represented. Several papers reported the isolation of multiple new, active compounds from two or more structure classes simultaneously (63, 7%). For biological activity, the focus of research was overwhelmingly on anti-bacterial (270) and cytotoxic activity (417), which were reported by numerous researchers (Figure 10f). However, reports on more specialized biological assays tended to be associated with particular research groups. For example, the majority of reports on alpha-glucosidase inhibitors (34 papers) were reported by Zhigang She and coworkers, while antifouling activity (32 papers) was a particular focus of Changyun Wang and co-workers in Qingdao. Likewise, most of the reports on anti-algal agents came out of the Chinese Academy of Sciences in Yantai. A large number of other biological activities were also reported in smaller numbers, often including the ability of compounds to inhibit enzyme systems, such as acetylcholinesterase, COX-2, or various kinases.

Overall, this review illustrates the diversity of research being conducted by China-based research in the field of marine microbial natural products drug discovery, while the increasing pace of publication suggests that China will continue to be a major player in the field over the years to come. 

## 9. Methodology

For the purposes of this review, only papers reporting the isolation of compounds with biological activity were reported. The scope of the review was limited to SCI-indexed journals. Only papers with at least one corresponding author based in China are included. Most of the papers reviewed reported active new compounds, but papers the reporting known compounds were also included, provided that the compounds possessed significant new activity. A full list of papers summarized in the review is given in Supplementary Table S1. For collection location, most of the categories are self-explanatory, however, ‘sediment’ encompassed both marine sediment, material from beaches, or even soil in a high salt environment (e.g., around mangroves). For biological activity: antioxidant activity also includes radical scavengers; anti-fouling activity incorporates biofilm inhibition; and “cytotoxic” includes compounds active against both cancerous and non-cancerous cell lines. Structural classifications are assigned for the new compounds isolated, or, if no new compounds were reported, for the active compounds. This was carried out by (a) accepting the classification of the author, if one was given, (b) checking if biosynthesis was known for the compound or structure class, or (c) careful observation of the structures. Heterologous expression studies, or compounds that were isolated from mutant strains produced by specific molecular manipulations were not included, but the mutant strains produced by other methods were included in the review.

## Supplementary Materials

The following are available online at https://www.mdpi.com/1660-3397/17/6/339/s1, Table S1 containing all references surveyed for the Discussion section of this review.
